# How Do University Students Perceive Inequality, Relationships and Power in University Life in the COVID-19 Era?

**DOI:** 10.3390/bs13090715

**Published:** 2023-08-28

**Authors:** Taeeun Shim, Songyi Lee, Mikyung Jun

**Affiliations:** 1 Student Competency Development Team, Dongguk University, 30, Pildong-ro 1 gil, Jung-gu, Seoul 04620, Republic of Korea; 2 Department of Counseling and Coaching, Graduate School, Dongguk University, 30, Pildong-ro 1 gil, Jung-gu, Seoul 04620, Republic of Korea; 3 Department of Home Economics Education, College of Education, Dongguk University, 30, Pildong-ro 1 gil, Jung-gu, Seoul 04620, Republic of Korea

**Keywords:** inequality, relationship, power structure, COVID-19, university students

## Abstract

This study examined university students’ perceptions of inequality, relationships and power following the COVID-19 outbreak. We used a qualitative research method, inductive content analysis (ICA), to analyse their perceptions of inequality in their personal life, insiders and outsiders that show superiority in relationships and people with strong and weak characteristics of power structures. We extracted superordinate concepts, such as those in the individual, interaction and social/environmental dimensions, as the perceptions of inequality, insiders and outsiders and people with strong and weak characteristics. First, we found that university students experience inequalities when they perceive that individuals must cope independently with changes brought about by COVID-19. Second, the results showed that individuals can become insiders or outsiders depending on how they act during the COVID-19 pandemic. Finally, we demonstrated that strong individuals are less affected by COVID-19-related changes than weak individuals. Therefore, improving university students’ quality of life requires plans based on the students’ perceptions of inequality.

## 1. Introduction

Researchers have long discussed the issue of social inequality among university students to improve their quality of life. Inequality is a concept that emphasises the unfair distribution of power and resources, ranging from ‘opportunities’ to ‘processes’ to ‘results’ [1,2]. Researchers have generally discussed inequity from an economic perspective [3]. However, we must examine university students’ quality of life from various viewpoints. In addition to the economic perspective, we can explore inequality based on opportunities and outcomes [4,5,6]. Equal opportunity refers to equal access to education and addresses inequality resulting from unequal interactions between regions, schools, teachers and students. South Korea has one of the highest rates of university enrolment and one of the highest levels of educational enthusiasm. This has led to the rise in private tutoring, which can be quite expensive. Students from families that can afford to pay for these tutors have better educational outcomes [7]. In producing educational achievements, equality in the most active sense allows for fairness in the results despite inherent inequality [6]. Inequality recognised by college students is similar to educational polarisation, educational gaps and educational alienation. As a whole, researchers continue to treat inequality as an important topic in the awareness of problems in education [2]. Factors of inequality can include a deep connection to unfair treatment, such as discrimination against a specific group and privilege for another group.

For university students, the meaning of inequality, as their ability to do what they want and convert efforts into rewards through fair competition, is limited [8]. Moreover, university inequality can manifest as different experiences for students from diverse social classes, through class interactions, friendships and extracurricular activities [9].

In particular, inequality regarding university students’ daily lives changed significantly following the COVID-19 outbreak. Amid the central government’s enforcement of social distancing, many students lost out on the chance to experience life in the physical environment of a university and to interact with classmates, seniors and teachers. This lack of physical contact may have deprived students of the opportunities afforded by attending university, thereby creating new inequality. Inequity has existed in different forms in places out of sight. For instance, educational inequality increased as the dropout rate of young students rose due to long school closures because of COVID-19, particularly among low-income and disadvantaged races [10].

Furthermore, after COVID-19, educational problems manifested differently across different economic levels and countries, even across different cities and provinces within a country [11]. Additionally, differences in barriers due to distance learning appeared as inequal in class achievements. For example, among the various obstacles, the largest obstacle was access to the internet and technology [12,13]. While COVID-19 changed college life, we realized the need to alleviate educational inequality through programmes to support the use of digital devices and foster self-directed learning under remote education conditions. In addition, the expansion of remote education due to COVID-19 changed the appearance of the educational environment. It went from typical university education lecture rooms and various cultural and leisure facilities in the school to an individual digital-device-based educational environment [5].

Three levels of digital-related inequality exist: digital access, digital skills and outcomes. It is unclear how the disparity between digital access and digital skills relates to ‘outcome inequality’; however, it is the latest theory to explain the processes that lead to social inequality [14]. Researchers coined the term ‘digital divide’ to describe the unequal access to technology between the rich and poor in the United States in the 1990s, after which the expression rapidly spread in discussions of global disparity. Researchers argued early on that this digital divide would worsen educational disparities [15], and the issue of inequality due to the digital divide following the outbreak of COVID-19 has been evident.

In addition, the COVID-19 outbreak has shifted the educational landscape from school to home, which may be problematic, as it reduces the role of schools in lessening inequality. According to Bayrakdar and Guveli [16], schools had played a vital role in providing equal opportunities, and because schools closed and family roles increased, the possibility of educational inequality also rose. Additionally, social distancing and closures led to a decrease in public activities while increasing time spent at home [17], mixing the responsibility for education between home and school, with the family affecting education more following the COVID-19 outbreak. In a family-oriented culture such as South Korea, parents significantly influence education, meaning that inequalities due to family backgrounds can become severe. However, studies of the effect of parental circumstances on educational inequality have obtained mixed results [18].

Moreover, changes in inequality are not limited to education; they also appear in human relationships among university students. For instance, South Korean university students express the English term ‘insider’, concerning human relationships, as ‘insa’, referring to people who get along well with others in a group, have many connections and are central to their group. On the other hand, ‘outsa’, an abbreviation for ‘outsider’, refers to people who do not fit into a group and are more solitary. An ‘insa’ with good interpersonal relationships adapts well to university life, but an ‘outsa’ experiences high levels of stress, depression and anxiety [19,20]. In addition, an ‘insa’ has access to resources and power in relationships, while an ‘outsa’ is weak and alienated. Therefore, it can be said that an ‘insa’ may have more opportunities in relationships than an ‘outsa’.

Notably, social media use was necessary for an ‘insa’ to develop human relationships even before the outbreak of COVID-19. Social networking services (SNSs) are highly effective communication means for forming, maintaining and developing networks to obtain social capital and strengthen social ties [21]. Nevertheless, before the COVID-19 outbreak, it was common for university students to invest their time in face-to-face human relationships—university students who had power in human relationships used SNSs subsidiarily. However, university students who lacked ability in human relationships predominantly used SNSs rather than face-to-face activities. Following the emergence of COVID-19, it became impossible for anyone to have in-person human interactions. Thus, we cannot know how the balance of power manifested in human relationships under these circumstances. However, humans can influence relationships while experiencing power imbalances. Of course, this is not invariable. Individuals become relatively weak with persons stronger than themselves and become stronger around weaker persons [22]. As such, the concept of inequality shows relative characteristics.

Following the outbreak of COVID-19, university students began to spend more time alone, and their responsibility for leisure and self-management increased. Unlike time spent attending classes at university, leisure and self-management take diverse forms depending on the student. Thus, individuals’ resources and environments have more of an effect on their free time and self-management. Additionally, COVID-19 altered the routines that people previously took for granted. For instance, people changed how they spent their spare time due to the perception that sports or cultural leisure increases the potential threat of spreading infectious diseases [23]. In such a society, the power experienced by an individual is not absolute but relatively changeable. An individual becomes relatively weak when they are with someone stronger than themself but becomes stronger when with someone who is less powerful than themself [22]. The concepts of ‘strong people’ and ‘weak people’ are commonly used in Korea to describe inequality in the structure of power and resources. In other words, people are categorised as strong or weak based on how much power and resources they have in society. As a result, the weak are more likely to experience inequality in society.

COVID-19 also significantly altered the external circumstances that allowed university students to become aware of inequality. However, there are few studies on how university students—the persons directly involved—approach such social disparities. According to Noh, Kwon and Shim [24], university students’ risk of anxiety and depression increases with their perceptions of social inequality. Furthermore, according to a study by Browman, Destin and Miele [25], perceptions of socioeconomic mobility worsen as perceptions of inequality increase. This means that how one perceives one’s socioeconomic status is more important than one’s actual socioeconomic status [26].

Without understanding the inequality and superiority in relationships and power perceived by university students, it is challenging to address inequality in the changing university environment. Therefore, this study uses inductive content analysis (ICA) to examine university students’ perceptions of inequality following the COVID-19 outbreak. ICA is appropriate for exploring vague phenomena and explaining the worldview behind anomalies rather than focusing on theories [27]. Therefore, in this study, we examine how college students perceive inequality in their personal lives, in their relationships and in the social context of power structures during COVID-19. The research questions are as follows.

**RQ1.** How do college students perceive inequality during COVID-19?
**RQ2.** How do college students perceive insiders and outsiders (superiority in relationships) during COVID-19?
**RQ3.** How do college students perceive people who have strong and weak characteristics (power structures) during COVID-19?

## 2. Materials and Methods

This study analysed university students’ perceptions of inequality using ICA, a qualitative research method that uses text-based data written as records or documents of language interactions [27]. ICA includes clear and comprehensive content analysis and can explain texts with visible content [28,29]. It is a process of inferring certain conclusions by carefully interpreting unstructured data and systematically and objectively identifying the data’s characteristics by reinterpreting the message implied by the data based on a particular analysis criterion or viewpoint. Since ICA is a type of qualitative analysis, it does not calculate frequency, but it is often accompanied by frequency when quantitative content analysis is desired [30].

The study procedure used open coding, which involved classifying the concepts or topics of the collected data. Then, we created categories from the data, including subcategories for all the divided content. Finally, we applied abstraction, extracting topics centred on the categorised data in order of precedence [29,31].

### 2.1. Study Subjects

To examine how university students perceive inequality and superiority in relationships and power in university life in the COVID-19 era, we conducted an online semi-structured questionnaire for 15 days using Google Surveys with 224 liberal arts students at a private university in South Korea’s capital.

Before administering the questionnaire, we explained the purpose of the study, and only students who agreed to participate responded to the questionnaire. In the end, 154 out of the 224 students responded.

We conducted our study in compliance with the Committee on Publication Ethics (COPE) and obtained informed consent forms from our participants. Table 1 shows the number of study subjects by major.

### 2.2. Study Procedure

We collected data through an open-ended questionnaire to achieve the study purpose via ICA. After transcribing all the collected data into an Excel sheet, researchers with knowledge of qualitative research identified the intents and meanings of students’ messages through several iterations of careful reading and reflective inquiry. All researchers participated in the inductive process of defining the subjects from the raw data. Additionally, to enhance the internal validity, the researchers conducted triangulation to agree on the dissenting parts of responses on the content by obtaining the frequencies and percentages of the classified data to evaluate their importance with the categorised content. From the data collection stage to the data analysis stage, the validity and reliability of the study were enhanced by minimising the bias of the researchers’ interpretations of the data and the error of interpretation through multi-angle analysis (triangulation) methods such as member checks, review among members and expert advice (peer debriefing). At this time, a qualitative analysis method was utilised to classify and derive components by comparing keywords and word counts in context within the review of research participants and members [27,32]. In this process, if two researchers had similar opinions and one had a different opinion, the researchers followed the opinions of the two, and if all three had different opinions, the content was analysed in consultation. In other words, all researchers conducted the inductive process of defining themes from the described raw data together to identify the intentions and meanings of the messages presented by the students. In addition, to enhance internal validity, the disagreements in the responses were categorised into frequencies and percentages by conducting triangulation, where meetings were continued until a consensus was reached [32].

The study’s procedure included open coding, creating categories and abstraction. First, we surveyed participants using Google Surveys with open-ended questions so that the subjects could freely write their thoughts concerning university student inequality. Questions included ‘What are some of the things you find frustrating about your college experience during COVID-19 and why?’, ‘What kind of inequalities are prevalent in university life during COVID-19 and why?’, ‘What kind of persons are insiders in university life during COVID-19 and why?’, ‘What kind of persons are outsiders in university life during COVID-19 and why?’ ‘What kind of persons are strong in university life during COVID-19 and why?’ and ‘What kind of persons are weak in university life during COVID-19 and why?’. Three researchers individually read the response data of the 154 subjects several times. They generated 823 open codes through discussion focusing on topics related to inequality: 159, 137, 218, 153 and 156 codes pertained to inequality, insiders, outsiders, strong people and weak people, respectively. Then, the researchers organised the codes with similar concepts into representative codes. For example, researchers re-coded ‘Differences in information acquisition’ and ‘Different amounts of information on personal connections’ into ‘Inequality of access to information’.

Second, the researchers grouped open codes with similar concepts to create subcategories. For example, researchers categorised ‘Inequality of access to information’ and ‘Differences in the amount of information obtained due to the number of personal connections’ into ‘Information inequality’. Thus, we categorised the codes as follows: inequality (n = 16), insiders (n = 9), outsiders (n = 10), strong people (n = 10) and weak people (n = 10). Finally, through the researchers’ agreement in the abstraction process, we extracted the personal dimension for oneself, the interaction dimension for relationships and communication with people and the social/environmental dimension for the surrounding environment.

## 3. Results

### 3.1. Meanings of Inequality

The results revealed that university students experience inequalities when they perceive that individuals must cope with COVID-19-related changes independently. For the personal dimension, university students indicated that inequality refers to being unable to experience university life (8, 5.0%), personality differences (3, 1.9%), deprivation of equal benefits (2, 1.3%) and a lack of a sense of belonging (2, 1.3%). For the interaction dimension, university students described inequality as information inequality (22, 13.8%), lack of opportunities to form relationships (9, 5.7%) and unavoidable circumstances due to others (1, 0.6%). Finally, for the social/environmental dimension, characteristics of inequality included the changing teaching environment (25, 15.7%), residential areas (24, 15.1%), class evaluations (19, 11.9%), tuition fees (15, 9.4%), internet devices (14, 8.8%), the need to incur additional expenses due to the changing environment (9, 5.7%), the inability to use the university environment (4, 2.5%), deterioration of the quality of education (1, 0.6%) and restrictions on face-to-face activities (1, 0.6%) (see Table 2).

The sudden changes in the university environment due to COVID-19 placed students in an unfamiliar context; some students quickly adapted, while others did not. Some existing resources prepared for university life became useless, demanding new abilities and amenities. University students had to adapt to the COVID-19-related changes individually. Some students perceived the changes in university life and the loss of aspects they had previously enjoyed as ‘deprivation’ and thus experienced a ‘lack of a sense of belonging’. They perceived non-face-to-face situations as preventing them from having opportunities to form relationships. Some responses included: ‘It is impossible to attend classes smoothly due to school policies that do not sufficiently reflect the diverse class environments’ (ID2), ‘The discomfort and difficulties caused by COVID-19 are felt as inequality’ (ID16). ‘Inequality was experienced when ‘poverty’ was connected to ‘difficulty accessing the server’ (ID30). Therefore, university students perceived inequality as the inability to access experiences and social resources that they had previously taken for granted due to COVID-19.

### 3.2. Meanings of Insiders and Outsiders

The results showed that individuals can become either insiders or outsiders depending on how they act during the COVID-19 pandemic. For the personal dimension, university students stated that insiders included those who are adaptable (15, 10.9%), are knowledgeable (2, 1.5%), excel at self-management (1, 0.7%) and maximally exhibit their abilities (1, 0.7%). For the interaction dimension, university students described insiders as persons who have relationship competence (29, 21.2%), forge non-face-to-face relationships (27, 19.7%) and possess existing relational resources (9, 6.6%). Finally, for the social/environmental dimension, insiders included persons who engage in external activities (27, 19.7%) and are involved in school activities (26, 19.0%).

For the personal dimension, university students characterised outsiders as less adaptable (20, 9.2%), clumsy when using media necessary for non-face-to-face activities (2, 0.9%), lacking goals (1, 0.5%) and having less personal time (1, 0.5%). For the interaction dimension, outsiders included those lacking the ability to form relationships (13, 16.5%), not using SNSs (8, 6.0%) and without human resources (8, 3.7%). Lastly, for the social/environmental dimension, outsiders’ characteristics included physical constraints (104, 47.7%), a lack of engagement in school activities (23, 10.6%) and a lack of involvement in external activities (10, 4.8%) (see Table 3).

The university students perceived that insiders exhibited adaptability despite sudden COVID-19-related changes, collected the necessary information and actively participated in school activities while effectively managing themselves. They defined them as ‘students with good sociality’ (ID78), ‘persons who are humorous and have good sociability’ (ID13, ID22) and ‘persons who make new friends’ (ID34). Another important characteristic of insiders was closely related to SNSs. Since social distancing measures made SNSs a vital communication tool, students considered ‘persons who communicate well with people through SNS’ insiders (ID64).

Outsiders were considered the opposite of insiders—students lacking relationship competence and who do not use SNSs. Outsiders were defined as ‘students who do not turn on the camera even when instructed by the professor during class’ (ID42), ‘passive and introverted’ (ID4), ‘students who do not speak even in group chat rooms’ (ID20), ‘shy persons’ (ID80) and so on. Interestingly, regarding insiders and outsiders, some claimed that the COVID-19-related changes made ‘most students … outsiders’ (ID2, ID49, ID138) because of how it significantly reduced university life, indicative of students’ reality in the COVID-19 era. The students stated that particularly first-year and rural students lacked human networks and became outsiders (ID149, ID150).

However, there was also a positive perception along with this pessimistic one. The students believed that individuals could become insiders or outsiders depending on their efforts to integrate into university life. Thus, the claims that ‘insiders before COVID-19 are insiders even in the COVID-19 era’ (ID15, ID32, ID105) and ‘if a person was an outsider before COVID, he should still be an outsider’ (ID65), indicating whether one is an insider or an outsider, largely depends on one’s personality and attitude. Thus, university students believed they could cope well with COVID-19-related changes through individual relationship competence and self-management. They also assumed that people were insiders or outsiders (indicating relational superiority) depending on their behaviours. We can see that the students’ perceptions of insiders and outsiders focus on their willingness and ability to relate to them.

### 3.3. Meanings of Strong and Weak People

The university students perceived that strong people were less affected by COVID-19-related changes than weak people and led lives similar to those they had lived before COVID-19. For the personal dimension, university students believed strong people to excel at self-management (45, 29.4%), information processing (20, 13.1%), achieving (10, 6.5%), being adaptable (7, 4.6%) and adapting to changes (4, 2.6%). For the interaction dimension, university students characterised the strong as persons with interpersonal skills (8, 5.2%). For the social/environmental dimension, university students believed strong individuals to be those who were free from physical environmental constraints (24, 15.7%), had economic resources (19, 12.4%), benefited from the changing class situation (8, 5.2%), had time resources (8, 5.2%) and so on (see Table 4).

For the personal dimension, university students believed weak people managed themselves poorly (27, 17.3%), had difficulty adapting (11, 7.1%), lacked information (10, 6.4%) and had magnified vulnerability (4, 2.6%). For the interaction dimension, university students considered weaker individuals to lack communication opportunities (7, 14.5%). Finally, for the social/environmental dimension, university students claimed the weak could not afford to purchase the equipment needed to adapt to the new environment (38, 24.4%), had physical limitations (25, 16.8%), lacked university experiences (21, 13.5%), were not guaranteed high-quality classes (10, 6.4%) and were not adaptable (3, 1.9%).

To university students, the strong included those whose lives appeared to be barely impacted by COVID-19: ‘those who can maintain the same activities as before’ (ID17) and ‘those who have nothing to do with changes in the environment’ (ID70). In addition, they thought that strong people could process information, self-manage, form relationships and have time and economic resources. They believed their capabilities and resources enabled them to adapt well to changes. The weak, however, were considered ‘persons who live in a rural area and do not have any smart device or independent space’ (ID76). Many university students defined higher achievers as strong (ID37, ID50, ID89, ID96, ID99 and ID106) and lower achievers and first-year students as weak (ID90, ID110, ID119, ID123 and ID149). They considered higher achievers to have more experience and human resources that helped them adapt to changes. In other words, their perceptions of the strong and the weak were based on how well they manage their personal lives based on the resources they have.

## 4. Discussion

This study examined how university students perceived inequality, relationships and power following the outbreak of COVID-19. Based on the findings of this study, we provide the following discussion points.

First, university students recognised the issue of educational inequality as crucial. They experienced inequality in the form of changed classroom environments, class evaluations, tuition fees, university environments and quality of education. This finding supports Choi’s [33] conclusion that university students perceive educational inequality regarding educational outcomes, curricula and opportunities.

In particular, post-COVID-19 educational inequality is closely related to digital-capital-based education. For instance, Andrew et al. [34] found that students’ access to digital data drove the educational attainment gap between children from poorer and wealthier families in the United Kingdom during COVID-19. In addition, Bayrakdar and Guveli [16] found that learning gaps occurred due to a lack of access to distance education during COVID-19, possibly due to difficulties accessing technology or insufficient parental support. Moreover, Blaskó et al. [35] and Frohn [36] found that the pandemic created a digital divide in performance. Gillis and Krull [12] also found that post-COVID-19 curriculum changes created barriers to internet and technology access, resulting in inequality for students. These studies support the findings of this study.

In South Korea, studies have shown that low-income children have less access to education, leading to differences in learning outcomes [37]. Along with access to education, we found that access to digital-based technology equipment is also critical. Furthermore, this study demonstrates the importance of inequality and the ability to use digital technology. This finding means that in addition to financial support, training in using these technologies is also essential in addressing educational inequality. Consequently, changes in the learning environment due to COVID-19 have made digital devices necessary for teaching and learning, and the ability to utilise digital devices has become an essential skill that determines learning ability [38]. These findings suggest that it is not only school authorities’ programmes but also instructors’ efforts to introduce these skills in each class that can help students develop digital competencies [39].

In addition, college students’ perceptions of inequality emphasised the physical environment. In particular, a prominent characteristic was that rural college students do not have easy access to urban universities. However, the notion of inequality due to place of residence predates COVID-19. According to Lee and Lee [40], the gap in self-confidence among young people by region in Korean society is very pronounced, with urban youth showing higher self-confidence than rural youth. Nevertheless, as the results of this study confirm, individuals characterise the concept of inequality in the post-COVID-19 era by experiences based on the physical environment. The future may bring situations, such as natural disasters, that force schools to switch to online classes. In such cases, universities need to have a system in place to open dormitories for rural students so that they do not experience double inequality.

Second, students’ perceptions of inequality between insiders and outsiders, emphasising the relational dimension, were reflected in the perceived importance of personal effort in relational competence. They showed that trying to build relationships, even if they are non-face-to-face, such as through social media, can make one an insider and thus empower one in relationships. Arslan, Yıldırım and Zangeneh [41] found that highly adaptive people have a higher sense of belonging in their relationships and express their identity strongly through social media use. The findings of this study suggest that dominance in interpersonal relationships also demonstrates the ability to adapt.

Hwang and Hwang [42] and Yoo and Woo [43] found that, unlike insiders, outsiders are less able to adapt to the environment and believe that they may be disadvantaged if others become aware of their problems. This conclusion supports the findings of this study that passivity in communication is the cause of inequality in relationships necessary for adaptation to the environment. In other words, it shows that the power structure in relationships does not depend on the use of SNSs but on how actively one can use communication methods according to the environment. Lee and Son [44] found that university-level support is needed to improve university students’ interpersonal skills in the post-COVID-19 era. The findings of this study provide evidence that marginalised groups, who are more likely to experience inequality, lack personal skills in relationships and therefore need support for relationship building at school.

Third, university students’ perceptions of who is strong and who is weak in the face of COVID-19 were mainly personal. This finding means that they viewed the unequal power structure created by COVID-19 as a function of individual attributes. They perceived inequality as dependent on individual efforts, such as self-management. Therefore, rather than viewing inequality as an external problem, they perceived it as an internal problem over which they had control.

One of the main reasons behind their perception of inequality as an internal problem is that they saw the pandemic as a problem for everyone, not just themselves. Thus, they wanted to lead their adaptation strategies during COVID-19 through internal changes rather than external ones. Korean university students believe in meritocracy. Since they experienced a system of rewards based on effort and ability [8], they see inequalities in power structures as something they can control through their actions.

Suggestions based on the findings of this study are as follows: Researchers stated that the COVID-19 era would strengthen the tendency towards individualised education and expand and deepen the educational gap between classes due to physical disconnection [3,45,46,47]. Additionally, researchers viewed inequality according to social structural factors such as income, power and knowledge following the COVID-19 outbreak, which exposed vulnerable social groups to more significant risks in the unequal social structure and extended the time required for recovery for these groups [48,49]. Nevertheless, university students may not be aware of this gap. As with the sudden implementation of social distancing following the outbreak of COVID-19 that limited the experience of university life, the lifting of social distancing measures now requires students to adapt to the in-person university environment, which has become another problem that individuals must address. Therefore, it is necessary to understand students’ academic capabilities in advance to reduce inequality caused by the educational gap.

In addition, the rapid external changes due to COVID-19 placed university students in a different environment and gave new meanings to their physical, mental and relational resources. Thus, utilising existing standards to understand university students may not help them lead successful university lives. For example, digital devices have become an essential learning tool, and digital literacy has become necessary since the COVID-19 outbreak. Specifically, there should be a scale for students’ digital competencies and a student-specific guide for students to improve their competencies in the classroom. This must be preceded by a foundation of digital competencies for educators. In addition, schools should open dormitories and create platforms to communicate with students, especially those who are physically disadvantaged, such as students living in rural areas. Therefore, researchers should use this study’s findings as a reference point to better understand students.

Although this study is meaningful, as it examined university students’ perceptions of inequality, relationships and power, it has several limitations. One limitation is that we did not conduct the study with students at universities in South Korea with diverse characteristics but focused on students taking a four-year university course in one city. That is, students at rural universities or two-year colleges may perceive inequality differently from students attending a four-year university in South Korea’s capital. Also, percentage breakdowns of the students can provide meaningful data for the result. Therefore, we recommend follow-up studies including percentage breakdowns of the students that show differences in perceptions of inequality based on university and geography characteristics.

Moreover, we did not analyse differences according to grade, gender or major. Findings on perceptions of inequality based on demographic backgrounds would contribute to reducing inequality for those with each characteristic. Accordingly, follow-up studies with quantitative approaches are necessary to discuss university students’ perceptions of inequality and prepare an additional basis for reducing experiences of inequality.

Furthermore, although there are seriously vulnerable groups among university students, we did not focus on those groups. As COVID-19 persisted, people began to experience mental health problems related to stress, anxiety and depression; these issues were more prevalent among low-income or vulnerable students [50]. Therefore, follow-up studies focusing on these groups of students are needed.

## 5. Conclusions

The study explored university students’ perceptions of inequality in their personal lives, in their relationships and in the social context of power structures during COVID-19. It was found that they recognised inequality in the individual, interaction and social/environmental dimensions. They believed that the educational gap arises due to personal digital devices and digital literacy and that having dominance in relationships and being powerful in the power structure depends on individual effort and will, so each dimension also emphasises individual will or effort as the cause of or solution to inequality. In addition, the university students thought that inequality in terms of residential areas significantly impacts the physical/environmental level. Therefore, school authorities should offer support in situations where it is difficult for individuals to resolve inequality. In terms of education, it is necessary to identify the digital capabilities of students and instructors and provide digital education tailored to their characteristics, and for students living in rural areas, it is necessary to take institutional measures, such as opening dormitories.

## Figures and Tables

**Table 1:** Characteristics of research subjects.

Major	M	F	T	1	2	3	4	T
College of Arts	4	8	12	9	0	2	1	12
College of Biosystems	0	1	1	0	1	0	0	1
College of Business Administration	7	9	16	7	5	1	3	16
College of Education	17	21	38	23	8	6	1	38
College of Engineering	12	11	23	8	2	10	3	23
College of Future Convergence	1	4	5	5	0	0	0	5
College of Law	0	1	1	0	0	1	0	1
College of Liberal Arts	6	5	11	0	4	3	4	11
College of Police and Justice	1	6	7	3	1	1	2	7
College of Social Sciences	12	28	40	28	4	4	4	40
Total	60	94	154	83	25	28	18	154

Note: M = male, F = female, T = total, 1 = first year, 2 = sophomore, 3 = junior and 4 = senior.

**Table 2:** Meanings of inequality.

Extraction of Meanings of Inequality	Primary Topics	Secondary Topics
Being unable to experience face-to-face university life (5) Being unable to participate in enjoyable university events (3)	Being unable to experience university life (8) (5.0%)	Personal dimension (15) (9.4%)
Differences caused by personality differences (2) Difference in feelings between those who want non-face-to-face interactions and those who do not (1)	Inequality due to personality differences (3) (1.9%)	
A feeling of not receiving equal opportunities compared to others (1) A difference in opportunities to experience university (1)	Deprivation of equal benefits (2) (1.3%)	
Lack of a sense of belonging due to a lack of human relationships (1) The state of being left alone without a sense of belonging (1)	Lack of a sense of belonging (2) (1.3%)	
Inequality of access to information (16) Differences in the amount of information obtained due to the number of personal connections (6)	Information inequality (22) (13.8%)	Interaction dimension (32) (20.1%)
Situations in which one cannot meet friends (6) Restrictions on face-to-face activities (3)	Lack of opportunities to form relationships (9) (5.7%)	
The negative effect on one’s exam results caused by the infection of a roommate with COVID-19 (1)	Unavoidable situations due to others (1) (0.6%)	
Inequality due to the difficulty of reflecting the characteristics of various people in non-face-to-face classes (12) Class type or academic environment that does not consider economic circumstances (6) Inability to practice (4) Deprivation of opportunities for quality education (3)	Inequality due to the changing classroom environment (25) (15.7%)	Social/environmental dimension (112) (70.4%)
Inequality in event participation due to differences in residential areas (14) Difficulty in forming friendships due to residing in a rural area (10)	Inequality due to residential areas (24) (15.1%)	
Cheating on exams (15) Feeling restricted in terms of testing location when sitting for an exam (4)	Inequality due to class evaluation (19) (11.9%)	
Lack of change in tuition fees despite COVID-19 (10) High tuition fees despite a lack of labs and practicals (5)	Inequality due to tuition fees (15) (9.4%)	
Situations where one should use a laptop despite not being proficient in using it (7) Gaps regarding access to the internet and information devices (7)	Inequality due to internet devices (14) (8.8%)	
A requirement to purchase goods to adapt to the changing environment (9)	Situations where additional expenses are incurred due to the changing environment (9) (5.7%)	
Being unable to use school facilities after paying tuition (4)	Being unable to utilise the university environment (4) (2.5%)	
Experiencing a decline in the quality of education due to non-face-to-face classes (1)	Deterioration of the quality of education (1) (0.6%)	
Restrictions on face-to-face activities (1)	Restrictions on face-to-face activities (1) (0.6%)	
Total	159 (100%)	159 (100%)

Note: Numbers in brackets = numbers of responses; percentages = percentages of total responses.

**Table 3:** Meanings of insiders and outsiders.

Extraction of Meanings of Insiders	Primary Topics for Insiders	Secondary Topics	Primary Topics for Outsiders	Extraction of Meanings of Outsiders
People who are interested in and passionate about university life (10) Self-confident persons (3) Leaders (2)	Persons with an adaptable personality (15) (10.9%)	Personal dimension (19/24) (13.9%/11.0%)	Persons who struggle to adapt due to their personality (20) (9.2%)	Persons who are very shy and timid (15) Persons who like staying at home (5)
Informative persons (2)	Informative persons (2) (1.5%)		Persons who are clumsy when using the media necessary for non-face-to-face activities (2) (0.9%)	Persons unfamiliar with machines (1) Persons who are not good at using computers and mobile phones (1)
Persons who excel at managing their lives (1)	Persons who excel at self-management (1) (0.7%)		Persons lacking goals (1) (0.5%)	Persons lacking goals (1)
Persons who can maximally use their abilities (1)	Persons who can maximally use their abilities (1) (0.7%)		Persons lacking personal time (1) (0.5%)	Persons who do not have time because of part-time jobs (1)
Persons who communicate well with others (15) Persons who try to meet new people (9) Persons who meet many friends (5)	Persons with relationship competence (29) (21.2%)	Interaction dimension (65/57) (47.4%/26.1%)	Persons who cannot form relationships (36) (16.5%)	Persons who do not try to get along well with others (17) Persons who do not make friends at university (13) Persons who do not participate in various small groups (6)
Persons who communicate well with people through SNSs (14) Persons who stay connected through mobile devices (8) Persons who often meet online (5)	Persons who forge non-face-to-face relationships (27) (19.7%)		Persons who do not use SNSs (13) (6.0%)	Persons who do not use SNSs (13)
Persons who have many friends (6) Persons who have many neighbourhood friends (3) Persons who know the professor (1)	Persons who have existing relationship resources (9) (6.6%)		Persons without human resources (8) (3.7%)	Persons who do not know any peers at their university (3) Persons lacking opportunities to meet seniors (3) First-year students without opportunities to chat with schoolmates (2)
Persons who participate in various external activities (27)	Persons who engage in external activities (27) (19.7%)	Social/environmental dimension (53/137) (38.7%/62.8%)	Persons with physical environmental constraints (104) (47.7%)	Persons who cannot come to school (98) Persons living in rural areas (6)
Persons who participate in school activities (21) Persons who are department representatives (3) Persons who work hard on group projects (2)	Persons who are involved in school activities (26) (19.0%)		Persons who do not engage in school activities (23) (10.6%)	Persons who do nothing at university (15) Persons who only take classes and do not participate in school activities (8)
			Persons who are not involved in external activities (10) (4.6%)	Persons who do not engage in any activities (6) Persons who often stay at home (4)
Total	137 (100%)		218 (100%)	

Note: Numbers in brackets = numbers of responses; percentages = percentages of total responses.

**Table 4:** Meanings of strong and weak people.

Extraction of Meanings of Strong People	Primary Topics for Strong People	Secondary Topics	Primary Topics for Weak People	Extraction of Meanings of Weak People
Persons who manage themselves well (38) Persons who adapt to non-face-to-face classes (7)	Persons who excel at self-management (45) (29.4%)	Personal dimension (86/51) (56.2%/33.3%)	Persons who struggle with self-management (27) (17.3%)	Persons who do not try (12) Persons who are poor at self-management (12) Persons lacking self-control (3)
Persons who utilise electronic devices diversely (12) Persons with an excellent ability to acquire information (6) Persons with strong IT skills (2)	Persons with information processing capabilities (20) (13.1%)		Persons who struggle with adapting due to their personality (11) (7.1%)	Passive and shy persons (5) Persons who enjoy face-to-face relationships (4) Persons who do not adapt to new situations (2)
Persons who enjoy university life (7) Persons who succeed in finding a job even in difficult times (2) Persons who achieve high grades through cyber lectures (1)	High achievers (10) (6.5%)		Persons who lack information (10) (6.4%)	Persons lacking digital skills (7) Persons lacking the computer skills necessary for classes (3)
Introverted persons (4) Independent persons (2) Persons with weak presentation skills (1)	Persons with an adaptable personality (7) (4.6%)		Persons with magnified vulnerability (4) (2.6%)	Disabled persons who are at a disadvantage during non-face-to-face classes (3) Students who have to come to school despite their fear of COVID-19 due to responsibilities and duties (1)
Persons who turn a crisis into an opportunity (2) Students who adapt well to the internet in the COVID-19 era (1) Persons who are active, as they were before COVID-19 (1)	Persons who are adaptable to change (4) (2.6%)			
Persons who foster relationships (5) Persons who are active in relationships (3)	Persons with interpersonal skills (8) (5.2%)	Interaction dimension (8/24) (5.2%/4.5%)	Persons lacking opportunities for communication (7) (14.5%)	Persons with few communication opportunities (5) Persons who cannot communicate with the professor (1) Persons who like relationships with people but cannot maintain them due to given circumstances (1)
Persons living near Seoul (16) Persons whose homes are far from the school (6) Job-seeking persons with fewer restrictions on the time they have available for job preparation (2)	Persons free from physical environmental constraints (24) (15.7%)	Social/environmental dimension (59/98) (38.6%/62.2%)	Persons who cannot afford to purchase the equipment necessary to adapt to the new environment (38) (24.4%)	Persons who struggle in online classes due to a lack of internet devices (20) Persons with a poor learning environment due to a lack of financial resources (18)
Persons who have no problems with their learning environment because they have sufficient funds (10) Persons who have various electronic devices (7) Persons who have secured a personal space (2)	Persons who are economically well-off (19) (12.4%)		Persons with physical limitations (25) (16.8%)	Persons residing in rural areas (25)
			Persons lacking university experiences (21) (13.5%)	First-year students who cannot enjoy university life (21)
Persons with higher grades, which is advantageous for credit management (5) Persons subject to an absolute grading system (3)	Persons who benefit from the changing class evaluation system (8) (5.2%)		Persons who are not guaranteed high-quality classes (10) (6.4%)	Persons who need to take practicals/labs (5) Persons who cannot participate in face-to-face lectures (5)
Persons who excel at time management (6) Persons who can save commuting time (2)	Persons with time resources (8) (5.2%)		Persons who struggle with adapting to the changing class evaluation system (3) (1.9%)	Persons who study hard but do not receive good grades due to the absolute grading system (2) Persons who can memorise well due to changes in the evaluation system (1)
Total	153 (100%)		156 (100%)	

Note: Numbers in brackets = numbers of responses; percentages = percentages of total responses.

## Data Availability

Data from the study are available upon request.
